# Identification and Functional Analysis of the *Ph-2* Gene Conferring Resistance to Late Blight (*Phytophthora infestans*) in Tomato

**DOI:** 10.3390/plants13243572

**Published:** 2024-12-21

**Authors:** Chunyang Pan, Xin Li, Xiaoxiao Lu, Junling Hu, Chen Zhang, Lianfeng Shi, Can Zhu, Yanmei Guo, Xiaoxuan Wang, Zejun Huang, Yongchen Du, Lei Liu, Junming Li

**Affiliations:** State Key Laboratory of Vegetable Biobreeding, Institute of Vegetables and Flowers, Chinese Academy of Agricultural Sciences, Beijing 100081, China; 82101211132@caas.cn (C.P.); lixin10@caas.cn (X.L.); luxiaoxiao914@163.com (X.L.); hujunling98678@163.com (J.H.); 82101212258@caas.cn (C.Z.); 82101222257@caas.cn (L.S.); zhucan@caas.cn (C.Z.); guoyanmei@caas.cn (Y.G.); wangxiaoxuan@caas.cn (X.W.); huangzejun@caas.cn (Z.H.); duyongchen@caas.cn (Y.D.)

**Keywords:** tomato, late blight, *Ph-2*, candidate gene, hypersensitive responses

## Abstract

Late blight is a destructive disease affecting tomato production. The identification and characterization of resistance (R) genes are critical for the breeding of late blight-resistant cultivars. The incompletely dominant gene *Ph-2* confers resistance against the race T_1_ of *Phytophthora infestans* in tomatoes. Herein, we identified *Solyc10g085460* (*RGA1*) as a candidate gene for *Ph-2* through the analysis of sequences and post-inoculation expression levels of genes located within the fine mapping interval. The *RGA1* was subsequently validated to be a *Ph-2* gene through targeted knockout and complementation analyses. It encodes a CC-NBS-LRR disease resistance protein, and transient expression assays conducted in the leaves of *Nicotiana benthamiana* indicate that *Ph-2* is predominantly localized within the nucleus. In comparison to its susceptible allele (*ph-2*), the transient expression of *Ph-2* can elicit hypersensitive responses (HR) in *N. benthamiana*, and subsequent investigations indicate that the structural integrity of the *Ph-2* protein is likely a requirement for inducing HR in this species. Furthermore, ethylene and salicylic acid hormonal signaling pathways may mediate the transmission of the *Ph-2* resistance signal, with *PR1*- and HR-related genes potentially involved in the *Ph-2*-mediated resistance. Our results could provide a theoretical foundation for the molecular breeding of tomato varieties resistant to late blight and offer valuable insights into elucidating the interaction mechanism between tomatoes and *P. infestans*.

## 1. Introduction

Tomato (*Solanum lycopersicum*) is among the most economically significant vegetables globally [1]. Late blight, caused by *P. infestans*, is a devastating disease that can manifest at all stages of tomato growth and development [2,3]. The annual production losses attributed to late blight, along with the associated expenditures on chemical fungicides for its control, collectively amount to billions of dollars [4,5,6,7,8,9]. Moreover, *P. infestans* exhibits significant evolutionary potential, and the emergence of fungicide-resistant strains necessitates a shift away from reliance on single chemical strategies for effective control [10,11]. Hence, the integration of late blight resistance genes into tomato varieties has become a primary focus in contemporary tomato breeding efforts [4].

In recent decades, several R genes (*Ph-1*, *Ph-2*, *Ph-3*, *Ph-4*, *Ph-5-1*, and *Ph-5-2*) and quantitative trait loci (QTLs) against late blight have been identified in wild tomato species [4,12,13,14,15,16,17,18,19,20,21,22]. Previous studies have demonstrated that tomatoes harboring the *Ph-2* and *Ph-3* genes exhibit superior resistance against most prevalent races and show the prospective in tomato breeding programs [2,4,18]. However, to date, only the *Ph-3* gene has been cloned, while information regarding the *Ph-2* gene remains limited [2,4,16]. The *Ph-2* gene is an incompletely dominant gene derived from *Solanum pimpinellifolium* West Virginia 700 (WV 700) and exhibits substantial resistance to the race T_1_ of *P. infestans* while effectively delaying disease onset [2]. In the previous study, the *Ph-2* gene was mapped to a region of approximately 141 kb at the terminus of chromosome 10 through the F_2_ segregating population derived from the resistant line LA3152 and susceptible line LA3988 hybridization [4]. Although several candidates, including one *CC-NBS-LRR* type gene, were proposed in that research, the *Ph-2* gene remains unidentified, and its resistance mechanism has not been comprehensively understood [4].

Consistent with the majority of cloned potato late blight resistance genes, the first cloned *Ph-3* gene in tomatoes also encodes an NBS-LRR protein (NLR) [16,23,24]. Generally, members of the *NBS-LRR* gene family encode proteins that exhibit a canonical modular structure comprising a nucleotide-binding site (NBS) and a leucine-rich repeat (LRR) domain. Based on the presence of toll/interleukin-1 receptor (TIR) or coiled-coil (CC) domains, NLRs can be classified into two subclasses: TIR-NB-LRR (TNL) and CC-NB-LRR (CNL) [25]. The central NBS domain of NLRs functions as a molecular switch that regulates protein activity by binding to and hydrolyzing nucleotides. Any mutation resulting in abnormal ADP binding or ATP hydrolysis capabilities within the NBS domain may disrupt its homeostasis and trigger an autoactivation state [26,27]. In this state, the persistent immune response can inhibit normal plant growth and development, leading to stunted stature, premature senescence, and reduced yield [28,29,30,31,32,33]. Typically, transitions in the state of the NBS domains are induced by conformational changes in the C-terminal LRR domains. Under the activated condition, the N-terminal domain of NLRs can bind to downstream signaling factors, thereby initiating the transmission of immune signals [28,29,34]. Relevant reports indicate that the C-terminal LRR domain plays a crucial role in recognizing effector proteins from pathogens [25,35,36]. Upon recognition, it undergoes conformational changes that initiate downstream signal transduction and HR [29]. Previous research has shown that LRR domains are generally more evolvable than the others, suggesting that this gene family may acquire new resistance by diversifying ligand perception during evolutionary processes [35,37,38,39,40,41].

In this study, the potential candidate genes were further analyzed through a comparative analysis of sequence variation and expression changes associated with disease resistance. The *Ph-2* gene was subsequently validated through complementation analysis and targeted knockout experiments. Additionally, the molecular mechanisms underlying the role of the *Ph-2* gene in conferring resistance to *P. infestans* were investigated and discussed.

## 2. Results

### 2.1. Sequence Analysis of Genes Residing Within the Candidate Interval

In a previous study, the *Ph-2* gene was localized to a region of approximately 141 kb on the long arm of chromosome 10 [4]. Searches conducted using the Solanaceae Genomics Network (SGN; https://solgenomics.net) revealed the presence of 21 genes within this region, including five putative resistance genes [4,42] (Table 1).

To screen for candidate genes within the candidate interval, we conducted an analysis of sequence variation utilizing the assembled high-quality genomes of susceptible genotype Moneymaker (MM, *ph-2*) and resistant genotype WV700 (*Ph-2*) [43]. The results revealed that, compared to MM, there are three genes with sequence variation in the exon region in WV700, which led to the change in related amino acids (Table 1). A sequence alignment revealed a total of 32 single-nucleotide polymorphisms (SNPs) in the first exon of the *Solyc10g085460* gene, resulting in 19 amino acid substitutions, while a single amino acid mutation was identified in each of the other two genes, *Solyc10g085510* and *Solyc10g085530*. Among the remaining 18 genes, mutations were identified located within introns in 6 genes, while no differences were observed in the sequences of the other 12 genes between the two genotypes. In addition, the CDS of the 21 genes was also verified through Sanger sequencing, and mutation analysis confirmed the presence of 32 SNP variations in the *Solyc10g085460* gene between resistant and susceptible genotypes. Notably, the coding sequences of the *Solyc10g085510*, *Solyc10g085520*, *Solyc10g085530*, *Solyc10g085540*, *Solyc10g085640*, and *Solyc10g085650* genes could not be successfully amplified via a polymerase chain reaction (PCR). Related studies suggest that this might be attributed to the absence of expression of these genes in leaves [42].

Furthermore, we conducted a comparative analysis of the promoter regions for these 21 genes between MM and WV 700, spanning approximately 3500 bp upstream of the coding sequences. The alignment results revealed that the promoter region of *Solyc10g085460* exhibited five mutations, while *Solyc10g085630* displayed three single-base substitutions. Additionally, the promoter regions of *Solyc10g085490* and *Solyc10g085500* each contained one base deletion and one single base substitution. The sequence alignment results of the promoter regions were further corroborated through Sanger sequencing (Table S1).

### 2.2. Analysis of Gene Expression Within the Candidate Interval

**Figure 1:**
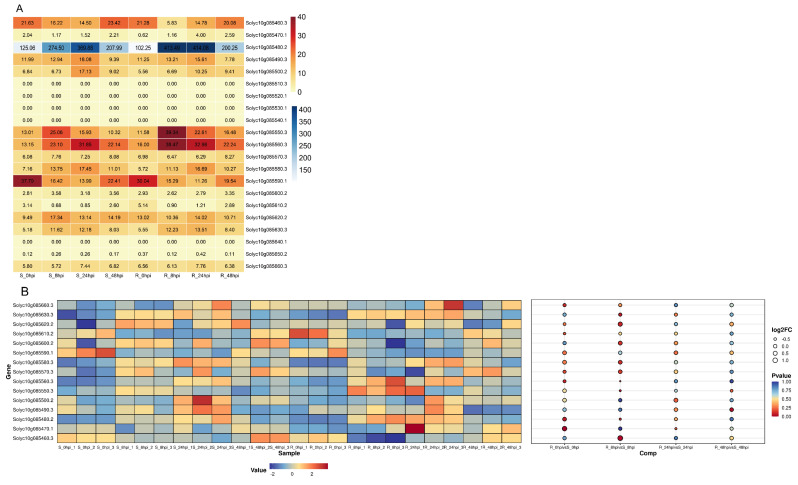
Analysis of gene expression within the candidate interval (**A**) Heatmap of expression of genes within the candidate interval after inoculation; (**B**) Heatmap–bubble of the 15 genes stably expressed in the candidate interval.

Based on the analysis of peroxidase activity and previous research, we propose that the *Ph-2*-mediated resistance response is activated within 48 hours post-inoculation (hpi) [44] (Figure S1). Considering the sequence variation in the promoter region and the absence of the expression of certain genes prior to inoculation with *P. infestans*, we conducted a post-inoculation transcriptome sequencing analysis on the *Ph-2* near-isogenic lines (NILs) to evaluate gene expression within the candidate intervals at four time points: 0 hpi, 8 hpi, 24 hpi, and 48 hpi (Figure S2). The expression heatmap of these 21 genes indicated that *Solyc10g085510*, *Solyc10g085520*, *Solyc10g085530*, *Solyc10g085540*, *Solyc10g085640*, and *Solyc10g085650* were either not expressed or exhibited very low levels of expression at the four sampling time points (Figure 1A). And the expression patterns of the remaining 15 genes were largely consistent between the resistant and susceptible genotypes (Table S2). Additionally, we conducted an analysis of differences in gene expression between the two genotypes at each time point, revealing that only the *Solyc10g085460* and *Solyc10g085550* genes exhibited significant differences at 8 hpi, while no other genes demonstrated significant differences post-inoculation (Figure 1B and Table S3).

### 2.3. Candidate of Ph-2 Gene

Through an expression analysis, six genes that were not expressed between 0 and 48 hpi were deemed less likely to be the *Ph-2* gene. Among the remaining 15 expressed genes, only *Solyc10g085460* and *Solyc10g085550* exhibited significant differences at 8 hpi between resistant and susceptible genotypes, while the promoter and gene sequences of *Solyc10g085550* gene displayed no differences between the two genotypes. Therefore, in conjunction with a coding sequence analysis, the *Solyc10g085460* gene was identified as the only gene expressed within the candidate interval with coding sequence variation. A functional annotation revealed that the *Solyc10g085460* gene encodes a CC-NBS-LRR disease-resistance protein. Consequently, we designated the *Solyc10g085460* (*RGA1*) gene as a candidate for the *Ph-2* gene.

### 2.4. Targeted Knockout and Complementation Analysis of the RGA1

**Figure 2:**
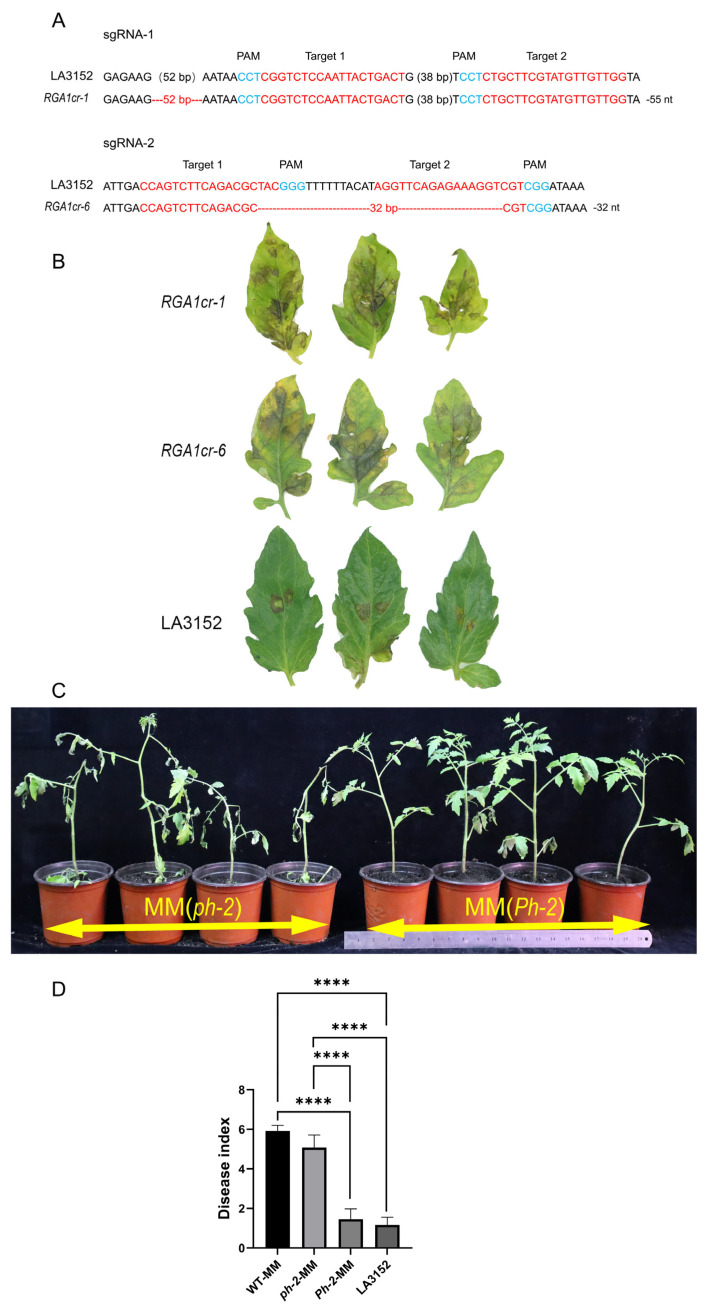
Targeted knockout and Complementation analysis of the *RGA1* (**A**) *RGA1* mutations generated through *CRISPR/Cas9* gene editing; (**B**) Comparison of phenotypes after inoculations between *RGA1cr-1*, *RGA1cr-6* and LA3152; (**C**) Comparison of phenotypes after inoculations between MM (*Ph-2*) and MM (*ph-2*); (**D**) Comparison of disease index after inoculations between resistant and susceptible genotypes. Asterisks indicate a significant difference (****, *p* < 0.0001).

To assess the potential of the *RGA1* gene in conferring resistance to race T_1_ in tomato, it was knocked out using the *CRISPR/Cas9* system. Four vectors were designed to disrupt the *RGA1* gene, targeting sites within the first exon (Figure 2A). This approach resulted in two null mutants of the *RGA1* gene: the *RGA1cr-1* mutant exhibited a 52 bp deletion, while *RGA1cr*-6 displayed a 32 bp deletion. The resistance characteristics of the *RGA1* knockout mutant and the wild type were evaluated by inoculating with the race T_1_, revealing that the knockout mutant was susceptible to race T_1_, while the wild type exhibited resistance following inoculation (Figure 2B).

Moreover, we selected fragments encompassing both the candidate gene and its allele, which consisted of 3846 bp of the full-length gene, 3301 bp upstream, and 2174 bp downstream. The two fragments were subsequently cloned into the expression vector pHaoNM to construct a functional complementary plasmid for *Agrobacterium tumefaciens*-mediated genetic transformation. Finally, eleven (resistant genotype of *Solyc10g085460*) and thirteen (susceptible genotype of *Solyc10g085460*) independent transformants of the susceptible genotype MM were obtained, respectively. Quantitative real-time polymerase chain reaction (qRT-PCR) analysis demonstrated that these transformants exhibited a stable expression of the introduced genes (Figure S3). The transformants were inoculated twice with race T_1_. The MM expressing the *RGA1* and the resistant genotype LA3152 (*Ph-2*) exhibited resistance to the disease post-inoculation, whereas the MM harboring the susceptible allele of *RGA1* displayed susceptibility (Figure 2C,D). Consequently, the *RGA1*, governed by its native promoter and terminator, was adequate to confer resistance against the race T_1_ in the susceptible MM, thereby confirming it as the *Ph-2* gene.

### 2.5. The 35S Promoter Is Capable of Driving the Transcriptional Expression of Ph-2

**Figure 3:**
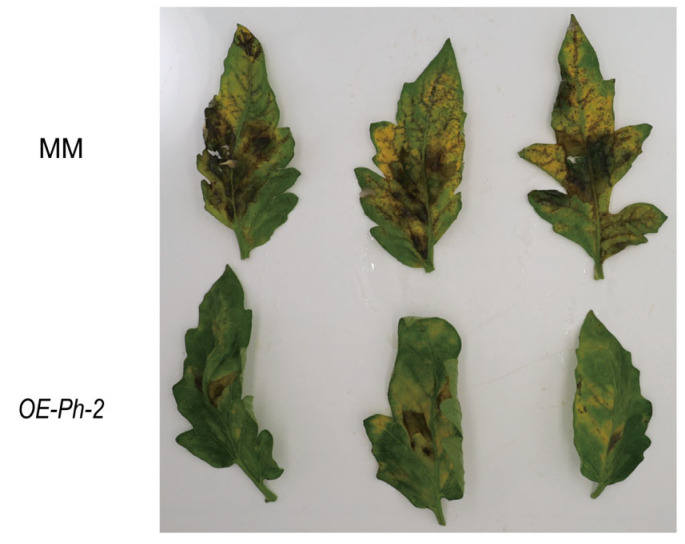
Identification of resistance to *Ph*-2 driven by the 35S promoter.

To determine whether *Ph-2* resistance is affected by its promoter sequence, we constructed an overexpression vector of 35S-*Ph-2*-HA for genetic transformation. Inoculation of the obtained overexpressed *Ph-2* MM showed that the expression of the *Ph-2* coding sequence could confer resistance to the MM (Figure 3).

### 2.6. The Subcellular Localization of Ph-2

**Figure 4:**
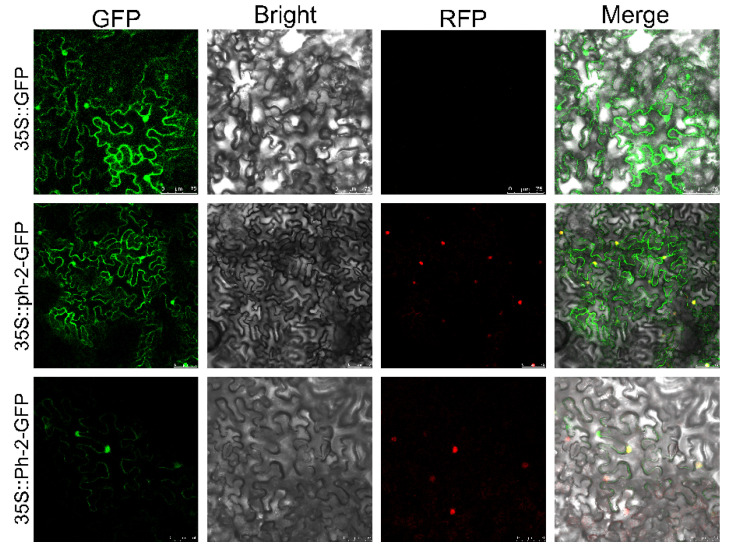
The subcellular localization of Ph-2 and ph-2.

Following the preliminary verification of the *Ph-2* gene’s function, we further investigated the characteristics and structure features of its encoded protein. Gene annotation revealed that the *Ph-2* gene encodes a CC-NBS-LRR resistance protein, with distinct domains of the protein serving varied functions in the activation and transduction of disease resistance signals [4,25]. Initially, the subcellular localization of the *Ph-2*/*ph-2* encoded protein was conducted by fusing a green fluorescent protein (GFP) tag to the C-terminus of the coding sequence. The transient expression of the Ph-2/ph-2 protein was conducted in *N. benthamiana* leaves under the control of the 35S promoter, while SV40 NLS-RFP was co-expressed as a localization marker. Under the confocal scanning laser microscopy, we observed that the ph-2 fluorescence signal was predominantly localized in the nucleus, with additional distribution on the cell membrane (Figure 4). A similar localization pattern was observed in Ph-2 expressing leaf cells; however, the fluorescence signal emitted by Ph-2 was significantly weaker than that produced by ph-2.

### 2.7. Evaluation of the Capacity of Ph-2 and Its Domains to Induce HR

**Figure 5:**
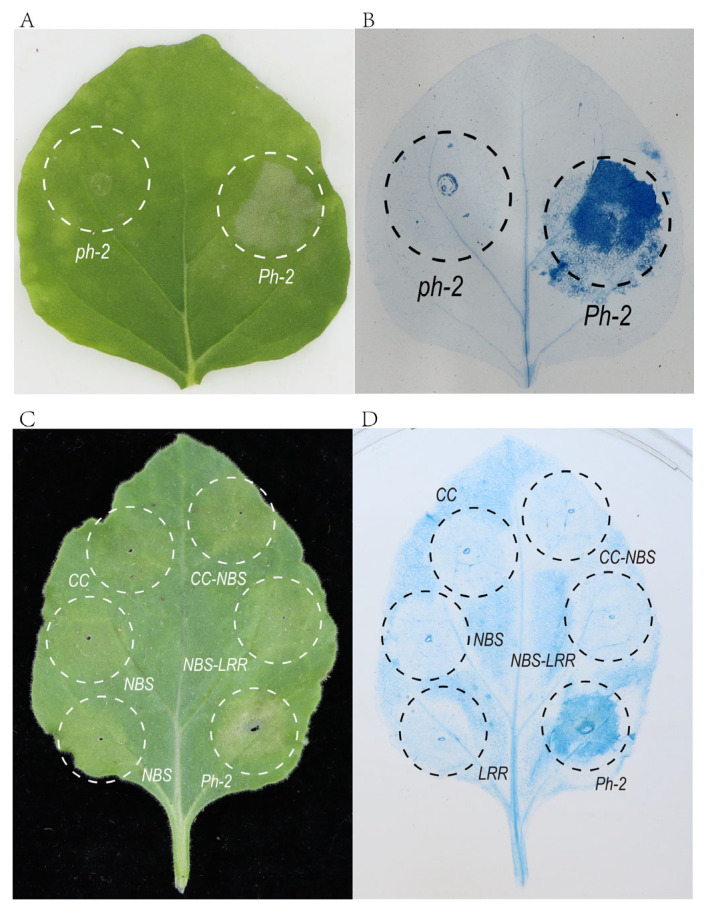
Evaluation of the capacity of Ph-2 and its domains to induce HR (**A**) HR response elicited by the transient expression of Ph-2; (**B**) Trypan blue staining; (**C**) Evaluation of the capacity of the Ph-2 domains to elicit HR; (**D**) Trypan blue staining.

In the Ph-2 subcellular localization assay, we observed suspected HR-induced cell death in the leaves of *N. benthamiana* transiently expressing *Ph-2*. It was clearly observed that the HR induced in the Ph-2 expressing position resulted in extensive cell death, whereas no HR was detected in the ph-2 expressing position (Figure 5A,B). This property of *Ph-2* is similar to that of reported auto-activated resistance genes [25,26,27,30,45]. The observed difference in this characteristic between ph-2 and Ph-2 indicates a substantial distinction between the two proteins concerning their resistance responses, which may account for their differing resistance to infection by *P. infestans*.

To identify the minimal Ph-2 domain required for HR induction, the structure of Ph-2 was divided into the CC domain, NBS domain, LRR domain, CC-NBS domain combination, and NBS-LRR domain combination [4] (Table S4). These segments and Ph-2 were fused to a yellow fluorescent protein (YFP) and transiently expressed in *N. benthamiana* leaves driven by the 35S promoter. Under the confocal scanning laser microscopy, we observed that the fluorescent signals from these five segments were present in the nuclear, plasma membrane, and cytoplasm (Figure S4). Observation of leaves transiently expressing these six components, along with trypan blue staining, revealed that HR was only observed at the site expressing intact Ph-2 (Figure 5C,D). This result indicates that an intact protein structure is necessary for Ph-2 to induce HR in *N. benthamiana* and that none of the other five individual domains or their combinations could induce the HR.

### 2.8. Comparative Transcriptome Analysis of Ph-2 NILs

**Figure 6:**
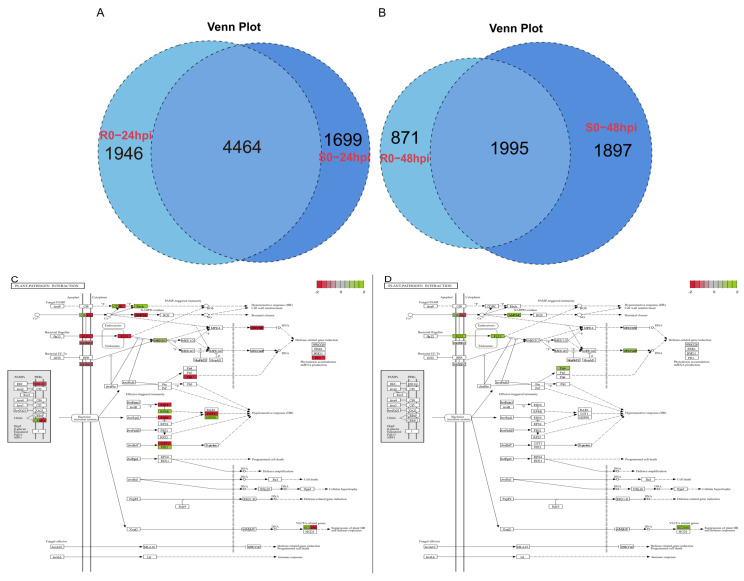
Comparative transcriptome analysis of NILs in *Ph-2* (**A**) The Venn diagram of DEGs of resistant and susceptible genotypes at 24 hpi; (**B**) The Venn diagram of DEGs of resistant and susceptible genotypes at 48 hpi; (**C**) The specific DEGs enriched in the ko04626 pathway at 48 hpi in the resistant genotype; (**D**) The specific DEGs enriched in the ko04626 pathway at 48 hpi in the susceptible genotype.

To investigate the mechanisms underlying *Ph-2*-mediated resistance, we conducted a comparative transcriptome analysis of its NILs. The analysis of the differentially expressed genes (DEGs) at 24 hpi and 48 hpi disclosed that the transcriptional levels of a considerable number of genes underwent modifications after inoculation (Table 2). The hormone signal transduction pathway ko04075 and the plant–pathogen interaction pathway ko04626 played a crucial role in plant resistance. The enrichment of DEGs in ko04075 indicated that following inoculation with *P. infestans*, the transcription levels of genes associated with ethylene and salicylic acid signaling pathways were significantly up-regulated in both *Ph-2* and its susceptible genotype, whereas those related to jasmonic acid signaling pathways were significantly down-regulated (Figure S5). The analysis of the plant–pathogen interaction pathway ko04626 revealed that both resistant and susceptible genotypes were capable of inducing the expression of genes related to HR and defense-related genes to varying degrees (Figure S6). Moreover, we conducted in-depth statistical and analytical operations on the DEGs of the two genotypes at 24 hpi and 48 hpi (Figure 6A,B) and found that the two genotypes possessed a considerable number of common DEGs at these two time points. To investigate whether the DEGs that differed between resistant and susceptible genotypes were related to resistance, we excluded the DEGs that were co-expressed and displayed the same trend in the two genotypes and subsequently performed KEGG pathway enrichment analysis of the different DEGs. The results indicated that at 48 hpi, a considerable number of HR- and disease-resistance-related genes were significantly up-regulated in the ko04626 pathway of the resistant genotype, whereas the expressions of related genes were significantly down-regulated in the susceptible genotype (Figure 6C,D). And similar findings were also obtained in the ko04075 pathway (Figure S7). Additionally, numerous DEGs related to plant–pathogen interactions, photosynthesis, and phenylpropanoid biosynthesis were enriched in the comparison between resistant and susceptible genotypes at 24 hpi and 48 hpi (Figure S8).

## 3. Discussion

In this study, we identified the *RGA1* gene as a potential candidate for *Ph-2* through the combination of sequence and expression analyses. Subsequent complementation analysis and targeted knockout experiments confirmed its ability to confer resistance in tomatoes against race T_1_, thereby establishing it as the *Ph-2* gene. The expression of the *Ph-2* gene driven by the 35S promoter can also confer resistance to late blight, suggesting that variations in its coding sequence may be critical for *Ph-2*-mediated resistance. The significant difference in *Ph-2* expression between resistant and susceptible genotypes at 8 hpi may be attributed to the transcriptional reprogramming initiated by surface-localized pattern recognition receptors (PRRs) during the early stages of pathogen infection [46,47]. The promoter and gene sequence of *Solyc10g085550* were completely consistent between resistant and susceptible genotypes. However, a significant difference was observed in the expression of this gene between the two genotypes at 8 hpi. This result indicates that transcriptional reprogramming responses differ between these two genotypes at 8 hpi, which may be associated with the distinct resistance pathways activated in each genotype.

The *Ph-2* gene encodes a CC-NBS-LRR disease-resistance protein. The 32 SNPs identified in the *Ph-2* gene between resistant and susceptible genotypes were all located within the coding region of the LRR domain [4] (Figure S9). Most known NBS-LRR resistance proteins are broadly distributed within cells, with their presence detected in the plasma membrane, nucleus, and cytoplasm; their distinct domains often occupy different cellular compartments [31,48,49,50]. This spatial distribution allows various domains to perform their respective functions; however, current studies indicate that the ultimate sites for executing resistance functions to induce immune responses are typically located in the nucleus [51]. In this study, we observed that the localization signal of the Ph-2/ph-2 protein predominantly resides in the nucleus, with a minor presence also detected at the plasma membrane, indicating that variations in the LRR domain between Ph-2 and ph-2 do not influence protein localization [52]. These findings suggest that the primary site for Ph-2′s recognition of pathogenic effectors may be situated in the nucleus, potentially resembling the action mechanism of Arabidopsis RRS1-R [52,53]. The C-terminal LRR domain of NBS-LRR resistance proteins frequently interacts with pathogen effectors and exhibits lower conservation compared to other domains [27,34,54,55]. And alterations in the LRR domains frequently lead to modifications in the resistance conferred by the protein [38,39,40,41]. Therefore, variations in the LRR domain of Ph-2 may alter its recognition of pathogenic effectors, potentially contributing to its resistance to race T_1_. The recognition of pathogenic effectors by the LRR domain often induces a conformational change that transmits the signal to the NBS domain. As the main domain of NBS-LRR proteins, the NBS domain contains eight highly conserved sequences: P-loop, RNBS-A, Kinase2, RNBS-B, RNBS-C, GLPL, RNBS-D, and MHDV [56]. These modifying mutations may result in the loss of self-repression of the gene, thereby triggering a series of negative effects and inducing HR in *N. benthamiana* [26,27,28,29,30,33]. When Ph-2 was transiently expressed in *N. benthamiana*, we observed that the site expressing Ph-2 exhibited an HR analogous to that of the auto-activated resistance protein, a phenomenon not observed at the site expressing ph-2. Although it has been reported that mutations in the LRR domain can lead to mismatching with the NBS domain, resulting in a loss of autorepression, no significant dwarfism or premature senescence was observed in MM lines expressing *Ph-2* [57,58]. Therefore, we propose that this phenomenon can only account for the differences in the characteristics of Ph-2 and ph-2 proteins, while the induction of HR by Ph-2 is more likely attributed to the overexpression or heterologous expression of resistance proteins.

With the exception of a few N-terminal domains of NBS-LRR proteins that can directly recognize pathogenic effectors, the majority of TIR/CC domains initiate the activation and transmission of disease resistance signals upon detecting alterations in the status of the NBS domain [59]. It has been reported that the signaling function of the CC domain is inhibited by the NBS domain and that expression of the CC domain alone is sufficient to induce an HR and activate the immune response [60]. Surprisingly, transient expression of the split Ph-2 and the combined Ph-2 domains did not elicit an HR; only the intact Ph-2 protein structure was capable of inducing HR. The current results do not elucidate whether this phenomenon is attributable to the characteristics of Ph-2 or to the lack of integrity in each domain when it is fragmented. These questions require further investigation in subsequent experiments.

Upon recognition of a pathogen, disease-resistance genes activate downstream immune responses that involve diverse hormonal signal transduction pathways [61]. Our transcriptome analysis indicated that ethylene and salicylic acid signaling pathways are implicated in the resistance signal transduction of tomatoes against *P. infestans*, which is consistent with the characteristics of resistance induced by this pathogen in other plant species [61,62,63]. Furthermore, during the early stages of infection, *PR1* and HR-related genes were induced in both resistant and susceptible genotypes. At 48 hpi, the expression of these genes was significantly down-regulated in the susceptible genotype while remaining persistently up-regulated in the *Ph-2* resistant genotype, indicating that the resistance response associated with *Ph-2* consistently activates the expression of related genes. At 24 hpi and 48 hpi, we observed a significant enrichment of DEGs related to disease resistance, further elucidating the differences in resistance pathways between the two genotypes. Regarding how Ph-2 interacts with downstream proteins to continuously activate these resistance genes, it will remain a key focus of our future research.

## 4. Materials and Methods

### 4.1. Plant Materials and Disease Assay

The *Ph-2* NILs utilized in this study, derived from the Heinz1706 background, were independently bred in our laboratory by using previously reported molecular markers as screening tools in conjunction with post-inoculation phenotypic selection [4]. The resistant line LA3152 (*Ph-2*) and susceptible line Moneymaker (accession number LA2706, *ph-2*) were kindly provided by the Tomato Genetics Resource Center (TGRC). Wild species *S. pimpinellifolium* West Virginia 700 was kindly provided by the Asian Vegetable Research and Development Centre (AVRDC).

The *P. infestans* race T_1_ isolate used as the inoculum can overcome the resistance mediated by *Ph-1* but not the resistance attributed to *Ph-2*. The isolate was maintained in 15% dimethyl sulfoxide solution at −80 °C and propagated on rye sucrose agar medium in the dark at 19 °C for 15–20 days before inoculation [63]. Plants with five fully expanded leaves were inoculated using a paint sprayer to disperse the suspension (1 × 10^4^ sporangia/mL) over the plants. Inoculated plants were incubated at 100% relative humidity (RH) and 20 ± 2 °C without light for the first 24 h. Thereafter, plants were grown at 70–90% RH and 20 ± 2 °C with a 12 h light period for 8 days [4]. Leaf samples were collected at 0, 8, 24, and 48 hpi. All samples were quickly frozen in liquid nitrogen and stored at −80 °C until DNA and RNA isolation.

Disease severity (DS) was rated 7 to 10 days after the inoculation using the following 0-to-6 scale: 0 = no apparent symptoms; 1 = 1 to 5% of the leaf area affected, with small lesions; 2 = 6 to 15% affected, with restricted lesions; 3 = 16 to 30% affected, with water-soaked flecks on the stem; 4 = 31 to 60% affected, with a few stem lesions; 5 = 61 to 90% affected, with expanding stem lesions; and 6 = 91 to 100% affected, with a severely damaged stem or dead plants. Plants with a DS score of 0 to 4 were considered resistant, whereas those with a DS score of 5 to 6 were designated as susceptible [63].

### 4.2. Sequence and Expression Analyses

DNA extraction of the samples was performed according to the method of Zhi et al. (2021) [4].

Total RNA extraction, cDNA synthesis, and qRT-PCR analysis were conducted following the methods previously described by Lu et al. (2021) [64] and Liu et al. (2019) [65]. All gene sequences within the candidate interval were retrieved from the Solanaceae Genomics Network (SGN; http://solgenomics.net). All primers were designed with the Primer 5.0 software (http://www.PremierBiosoft.com). PCR products were sequenced by Sangon Biotech Co., Ltd. Sequences were aligned using Snapgene 4.3.6 (https://www.snapgene.com/). All primers used for these analyses are detailed in Table S5.

### 4.3. RNA-Seq Analysis

A total of 24 RNA-Seq libraries, constructed with RNA from the *Ph-2* NILs, were sequenced using the Illumina NovaSeq6000 platform at Biomarker Technologies Co., Ltd. (Beijing, China). The clean reads obtained were aligned and quantified against the tomato genome (ITAG4.00) using HISAT2 version 2.1.0 [66] and String Tie version 2.0.6 [67]. Subsequently, gene expression levels were estimated with fragments per kilobase of transcript per million mapped reads (FPKM). We used DESeq2 [68] to detect differential gene expression between resistant material and susceptible material with criteria of a *p*-value ≤ 0.05 and |log_2_FoldChange| ≥1.00. And KOBAS 3.0 software was used to test the statistical enrichment of differential expression genes in KEGG pathways [69]. Heatmap–bubble plot and advanced Heatmap Plots were performed using the OmicStudio tools (https://www.omicstudio.cn/tool, accessed on 1 October 2024).

### 4.4. Vector Construction and Plant Transformation

DNA templates from LA3152 and Moneymaker were utilized to amplify a 9339 bp sequence, respectively, encompassing the promoter region, stop region, and full length of the *Solyc10g085460* gene. The sequence was subsequently cloned into the vector pHaoNM as the functionally complementary vector following the method described by Lu et al. (2021) [64]. For the CRISPR/Cas9 constructs, four sgRNA binding sites per vector were identified using CRISPR-GE (http://skl.scau.edu.cn/). The PCR fragments were then assembled into the binary vector pMGET through the Golden Gate cloning method, following the protocols described by Lu et al. too [64]. The plasmids mediated by *A*. *tumefaciens* strain GV3101 were individually transformed into the Moneymaker and LA3152 cultivars, following the methodology described by Sharma et al. (2009) [70]. cDNA from Moneymaker and LA3152 was utilized as a template to amplify the coding sequences of *ph-2*, *Ph-2,* and its corresponding domains. These fragments were subsequently cloned into the vectors pCAMBIA2300_35S_GFP-HA and pCAMBIA2300_35S_YFP-HA, respectively. And ATG was appended to the 5′ end of certain sequences that lacked a start codon. The previously mentioned target fragments were subjected to Sanger sequencing to verify their sequence accuracy. All primers used for these analyses are detailed in Table S5.

### 4.5. Subcellular Localization

According to the method of Wang et al. (2023) [71], all subcellular localization vectors were transformed into *A*. *tumefaciens* strain EHA105 and then co-infiltrated into the leaves of 4-week-old *N. benthamiana* plants together with RNA silencing suppressor P19 [72]. The localization of the fluorescent fusion proteins was analyzed using confocal scanning laser microscopy (Leica TCS SP8, Leica Biosystems, Weztlar, Germany). For YFP, we captured the images with laser excitation at 514 nm and collection bandwidth range from 525 to 570 nm. For GFP, we used 484 nm for excitation and collection bandwidth of 507 to 545 nm. For RFP, we used 514 nm for excitation and collection bandwidth of 585 to 625 nm. In all these experiments, the Gain did not exceed 600, and intensity values of about 5.45.

### 4.6. Trypan Blue Staining

Cell death in plant tissues was detected by trypan blue staining, as described previously by Bowling et al. (1997) [73]. In brief, plant tissues were submerged in a 65 °C trypan blue solution (2.5 mg of trypan blue per mL, 25% [*w*/*v*] lactic acid, 25% water-saturated phenol, and 25% glycerol) and heated over boiling water for 5 min and left to stain overnight. After destaining in chloral hydrate solution (2.5 g/mL) for 4 days, samples were equilibrated with 70% glycerol for photography (EOS 90D, Canon Inc., Tokyo, Japan) and microscopy (Stemi 2000-C, Carl Zeiss AG, Oberkochen, Germany) analysis.

### 4.7. Data Statistical Analysis

All relevant numerical calculations were used IBM SPSS Statistics 20.0 software in this study. Significance analyses of differences were conducted using a *t*-test to assess the results.

## 5. Conclusions

In conclusion, this study demonstrated that *RGA1*, which encodes the NBS-LRR resistance protein, is the *Ph-2* gene. The subcellular localization of *Ph-2* is primarily in the nucleus, with a minor distribution on the cell membrane. The transient expression of *Ph-2* was capable of triggering HR responses in *N. benthamiana* leaves. Transcriptome analysis indicated that ethylene and salicylic acid signal transduction pathways might mediate the signal transduction of late blight resistance in tomatoes, and *PR1*- and HR-related genes played a significant role in the *Ph-2* gene-mediated late blight resistance. The findings provide a theoretical foundation for breeding tomato varieties resistant to late blight by utilizing a combination of multiple genes and establishing a basis for elucidating the resistance mechanism of *Ph-2* against late blight.

## Figures and Tables

**Table 1:** Sequence analysis and annotation of the genes located within the candidate interval.

Gene	Position of the Mutation (5′ to 3′) and Type of Mutation	Annotation
*Solyc10g085460.3*	2612 bp (exon, T→G, Ile→Arg); 2617 bp (exon, G→A, Glu→Lys); 2640 bp (exon, A→G, Pro→Pro); 2666 bp (exon, T→C, Ile→Thr); 2673 bp (exon, T→C, Leu→Leu); 2676 bp (exon, G→A, Val→Val); 2677 bp (exon, A→G, Lys→Glu); 2683 bp (exon, GA→AG, Asp→Ser); 2688 bp (exon, T→C, Val→Val); 2689 bp (exon, G→T, Asp→Tyr); 2692 bp (exon, A→G, Asn→Asp); 2713 bp (exon, C→T, Leu→Phe); 2716 bp (exon, T→C, Ser→Pro); 2723 bp (exon, TT→AG, Val→Glu); 2727 bp (exon, G→T, Met→Ile); 2739 bp (exon, T→C, Asn→Asn); 2740 bp (exon, GTT→ATA, Val→Ile); 2752 bp (exon, TAC→GGA, Tyr→Gly); 2755 bp (exon, A→C, Ile→Leu); 2758 bp (exon, T→A, Ser→Thr); 2764 bp (exon, GTC→TCA, Val→Ser); 2768 bp (exon, A→G, His→Gly); 2772 bp (exon, T→C, Cys→Cys); 2773 bp (exon, G→A, Gly→Arg); 3298 bp (exon, G→T, Asp→Tyr)	CC-NBS-LRR resistance protein
*Solyc10g085470.1*	None	Unknown Protein
*Solyc10g085480.2*	None	60S ribosomal protein L24
*Solyc10g085490.3*	None	F-box protein PP2-B1
*Solyc10g085500.2*	936 bp (intron, T→A)	Cytochrome P450
*Solyc10g085510.3*	547 bp (exon, G→C, Ala→Pro)	Subtilisin-like protease
*Solyc10g085520.1*	None	Subtilisin-like protease
*Solyc10g085530.1*	428 bp (exon, A→G, Asp→Gly)	Subtilisin-like protease
*Solyc10g085540.1*	None	Subtilisin-like protease
*Solyc10g085550.3*	None	Enolase
*Solyc10g085560.3*	None	Histone deacetylase 2a-like
*Solyc10g085570.3*	5586 bp (intron, +CC); 5717 bp (intron, T→C); 10,796 bp (intron, +CC)	CTR1-like protein kinase-4
*Solyc10g085580.3*	245 bp (intron, +A)	Nucleolar protein 10
*Solyc10g085590.1*	None	Tumor susceptibility protein 101
*Solyc10g085600.2*	None	F-box/LRR-repeat protein 4
*Solyc10g085610.2*	None	Calmodulin binding protein
*Solyc10g085620.2*	None	Myb family transcription factor-like
*Solyc10g085630.3*	8163 bp (intron, +TT); 8330 bp (intron, +TATA)	Fatty acid oxidation complex subunit alpha
*Solyc10g085640.1*	279 bp (intron, + ATATAT)	Beta-fructofuranosidase insoluble isoenzyme 2
*Solyc10g085650.2*	None	Beta-fructofuranosidase insoluble isoenzyme 2
*Solyc10g085660.3*	954 bp (intron, G→A); 1210 bp (intron, -CCCC)	F-box protein SKIP24

**Table 2:** The statistical analysis of DEGs.

Comparative Groupings	All DEGs	Up-Regulated DEGs	Down-Regulated DEGs
R0 hpi vs. R24 hpi	6410	3174	3236
R0 hpi vs. R48 hpi	3892	2266	1626
S0 hpi vs. S24 hpi	6163	3469	2694
S0 hpi vs. S48 hpi	2866	1315	1551

## Data Availability

The RNA sequencing datasets generated in this study have been deposited in the Sequence Read Archive (SRA) under the accession number PRJNA1182783.

## Supplementary Materials

The following supporting information can be downloaded at https://www.mdpi.com/article/10.3390/plants13243572/s1, Table S1. Promoter sequence analysis of the expressed genes within the candidate interval.; Table S2. Gene expression trends within candidate intervals; Table S3. Significance analysis of differential gene expression within candidate intervals; Table S4 Gene sequences and promoter sequences; Table S5 Primers used in this study. Gene sequences and promoter sequences; Table S6 FPKM of all genes from the RNA-seq data; Table S7. RNA-seq data and statistical tables of DEGs. Figure S1. Analysis of peroxidase activity following inoculation with *P. infestans*; Figure S2. Correlation analysis of transcriptome samples; Figure S3. The relative expression of the *Ph-2* gene in transformants; Figure S4. Subcellular localization of the Ph-2 domains; Figure S5. Enrichment analysis of DEGs in the ko04075 pathway. (A) The DEGs enriched in the ko04075 pathway between R0 hpi and 24 hpi; (B) The DEGs enriched in the ko04075 pathway between R0 hpi and 48 hpi; (C) The DEGs enriched in the ko04075 pathway between S0 hpi and 24 hpi; (D) The DEGs enriched in the ko04075 pathway between S0 hpi and 48 hpi; Figure S6. Enrichment analysis of DEGs in the ko04626 pathway. (A) The DEGs enriched in the ko04626 pathway between R0 hpi and 24 hpi; (B) The DEGs enriched in the ko04626 pathway between R0 hpi and 48 hpi; (C) The DEGs enriched in the ko04626 pathway between S0 hpi and 24 hpi; (D) The DEGs enriched in the ko04626 pathway between S0 hpi and 48 hpi; Figure S7. Enrichment analysis of the DEGs specifically expressed differentially between resistant genotype and susceptible genotype within the ko04075 pathway. (A) Enrichment analysis of the DEGs specifically expressed differentially in resistant genotypes within the ko04075 pathway; (B) Enrichment analysis of the DEGs specifically expressed differentially in susceptible genotypes within the ko04075 pathway; Figure S8. KEGG enrichment analysis of DEGs between resistant and susceptible genotypes. (A): KEGG enrichment analysis of DEGs between resistant and susceptible genotypes at 24 hpi. (B): KEGG enrichment analysis of DEGs between resistant and susceptible genotypes at 48 hpi; Figure S9. Structural characteristics and amino acid sequence of Ph-2. (A) Predicted structure of the protein Ph-2; (B)The alignment of the amino acid sequence between Ph-2 and ph-2.
