# Supplementary material for: Identification and Functional Analysis of the Ph-2 Gene Conferring Resistance to Late Blight (Phytophthora infestans) in Tomato
Source: Plants (Basel). 2024 Dec 21;13(24):3572. doi: 10.3390/plants13243572 (PMC11679936; doi:10.3390/plants13243572)
Supplement: Supplementary file 1 [file plants-13-03572-s001.zip › Table S1&FigureS1-S9.pdf]

Table S1. Promoter sequence analysis of the expressed genes within the candidate interval.

Gene	Position of the mutation (3' to 5') and Type of mutation	Alignment of promoter sequences by Sanger sequencing
Solyc10g085460.3	804 bp (G→A); 3406 bp (+T); 3460 bp（-AAT）;3467 bp (T→C);3478 bp (-A)	804 bp (G→A); 3406 bp (+T); 3460 bp（-AAT）;3467 bp (T→C);3478 bp (-A)
Solyc10g085470.1	None	None
Solyc10g085480.2	None	None
Solyc10g085490.3	968 bp (-T) ;1741 bp (A→G)	968 bp（-T）;1741 bp (A→G)
Solyc10g085500.2	1396 bp (-T);2976 bp (A→T)	1395 bp (-T) ;2976 bp (A→T)
Solyc10g085550.3	None	None
Solyc10g085560.3	None	None
Solyc10g085570.3	None	None
Solyc10g085580.3	None	None
Solyc10g085590.1	None	None
Solyc10g085600.2	None	None
Solyc10g085610.2	None	None
Solyc10g085620.2	None	None
Solyc10g085630.3	442 bp（T→A）;700 bp (A→G);2687 bp (A→G)	442 bp（T→A）;700 bp (A→G);2687 bp (A→G)
Solyc10g085660.3	None	None

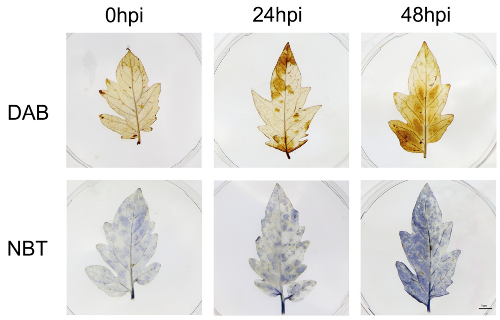

Figure S1. Analysis of peroxidase activity following inoculation with *Phytophthora infestans*.

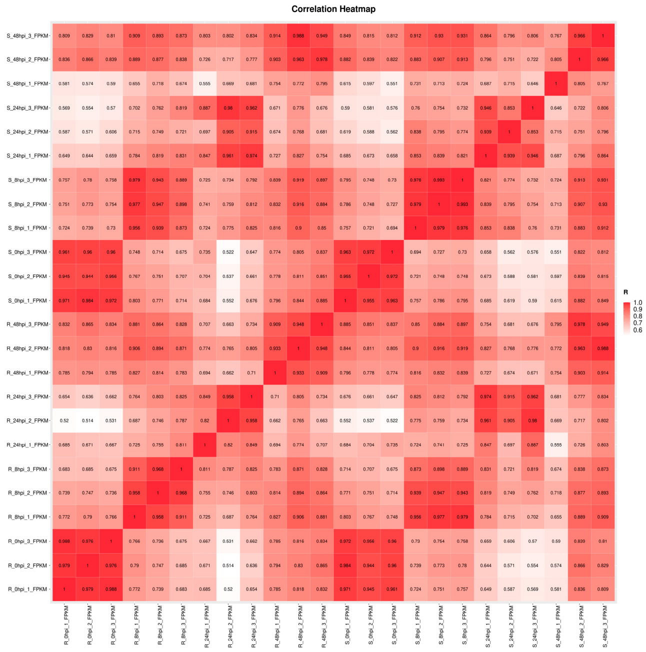

Figure S2. Correlation analysis of transcriptome samples.

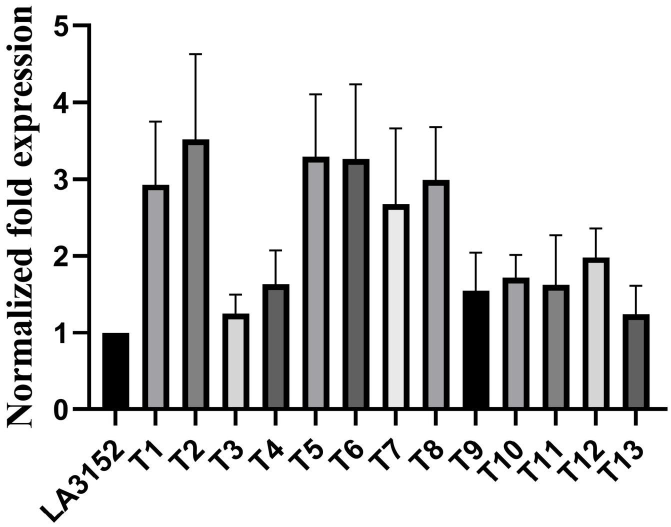

Figure S3. The relative expression of the *Ph-2* gene in transformants.

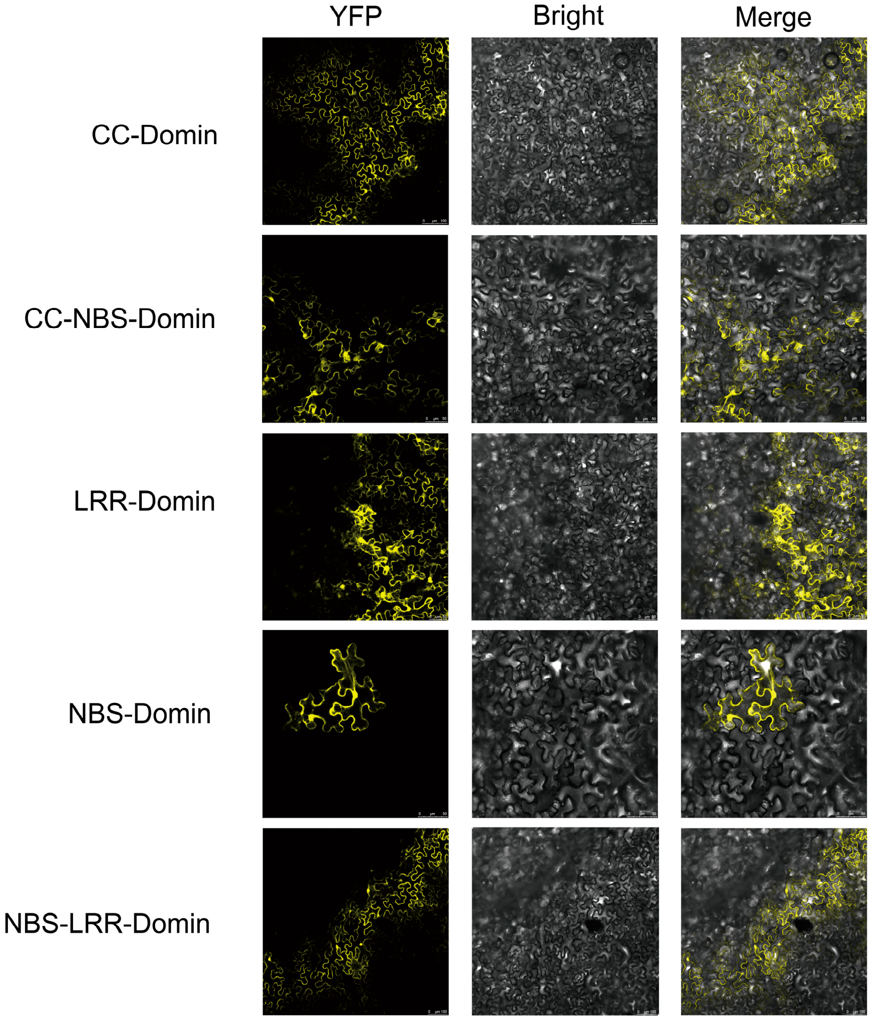

Figure S4. Subcellular localization of the Ph-2 domains.

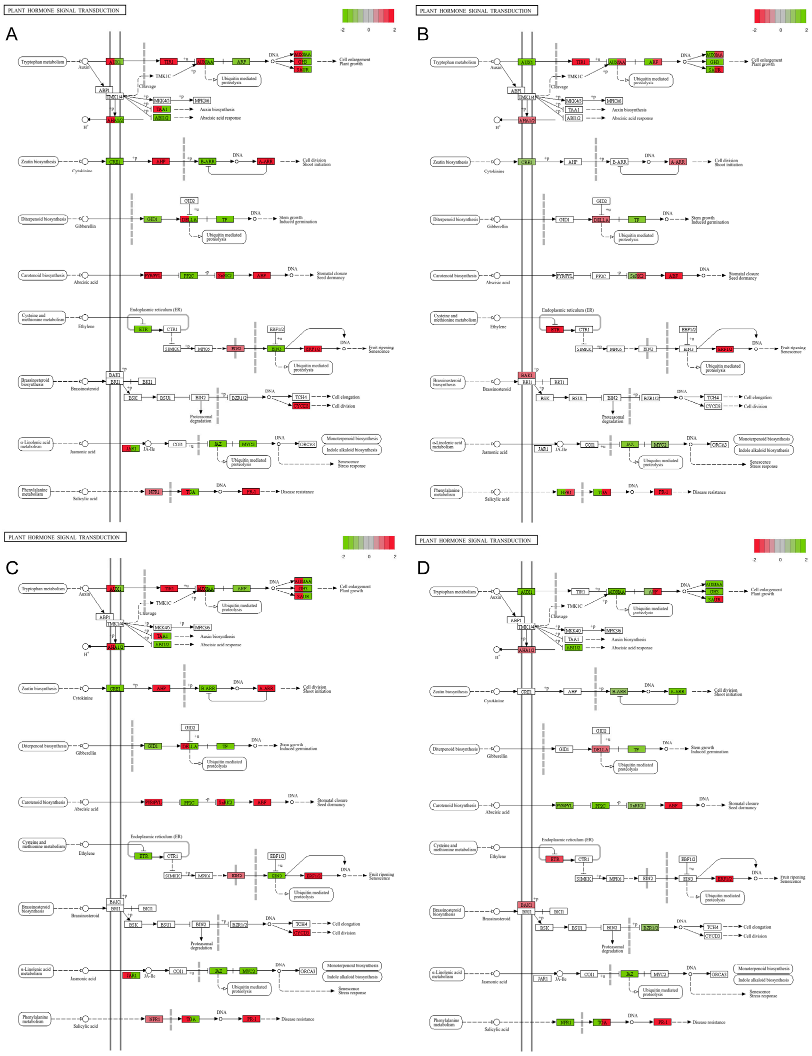

Figure S5. Enrichment analysis of DEGs in the ko04075 pathway. (A) The DEGs enriched in the ko04075 pathway between R0hpi and 24hpi;(B) The DEGs enriched in the ko04075 pathway between R0hpi and 48hpi; (C) The DEGs enriched in the ko04075 pathway between S0hpi and 24hpi; (D) The DEGs enriched in the ko04075 pathway between S0hpi and 48hpi.

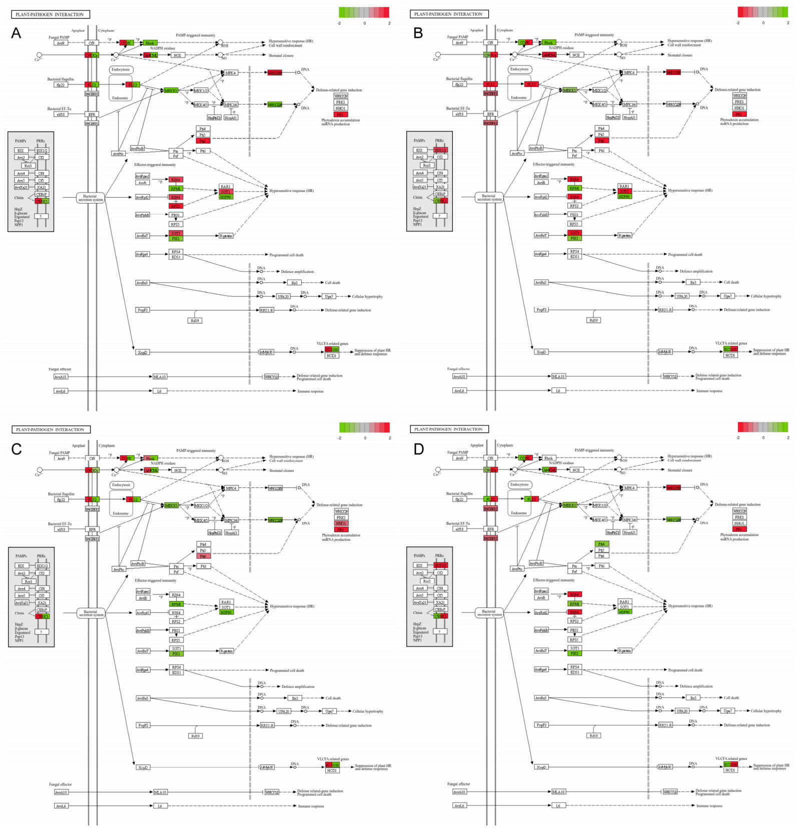

Figure S6. Enrichment analysis of DEGs in the ko04626 pathway. (A) The DEGs enriched in the ko04626 pathway between R0hpi and 24hpi;(B) The DEGs enriched in the ko04626 pathway between R0hpi and 48hpi; (C) The DEGs enriched in the ko04626 pathway between S0hpi and 24hpi; (D) The DEGs enriched in the ko04626 pathway between S0hpi and 48hpi.

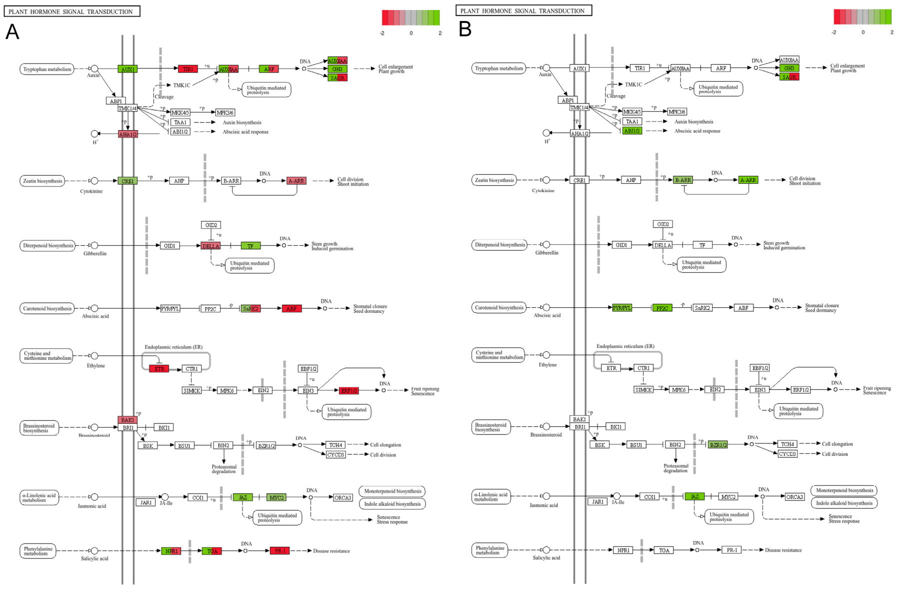

Figure S7. Enrichment analysis of the DEGs specifically expressed differentially between resistant genotype and susceptible genotype within the ko04075 pathway. (A) Enrichment analysis of the DEGs specifically expressed differentially in resistant genotypes within the

ko04075 pathway;(B) Enrichment analysis of the DEGs specifically expressed differentially in susceptible genotypes within the ko04075 pathway.

A

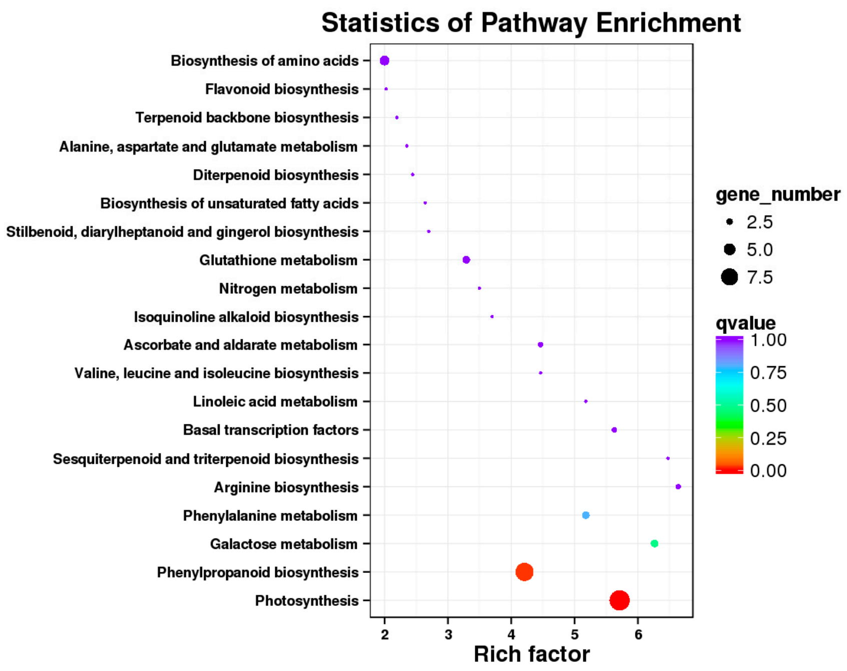

B

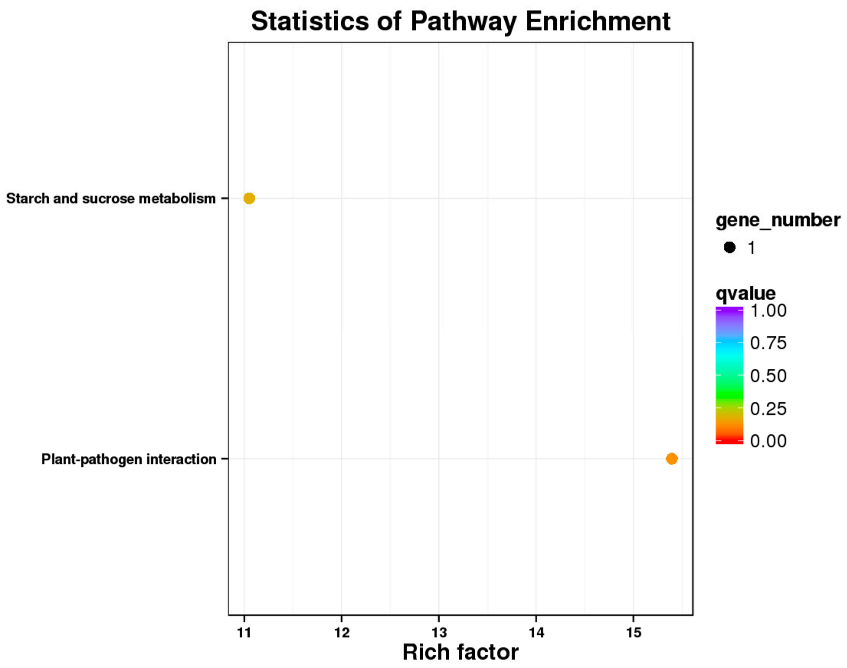

Figure S8. KEGG enrichment analysis of DEGs between resistant and susceptible genotypes. (A): KEGG enrichment analysis of DEGs between resistant and susceptible genotypes at 24hpi. (B): KEGG enrichment analysis of DEGs between resistant and susceptible genotypes at 48hpi.

A

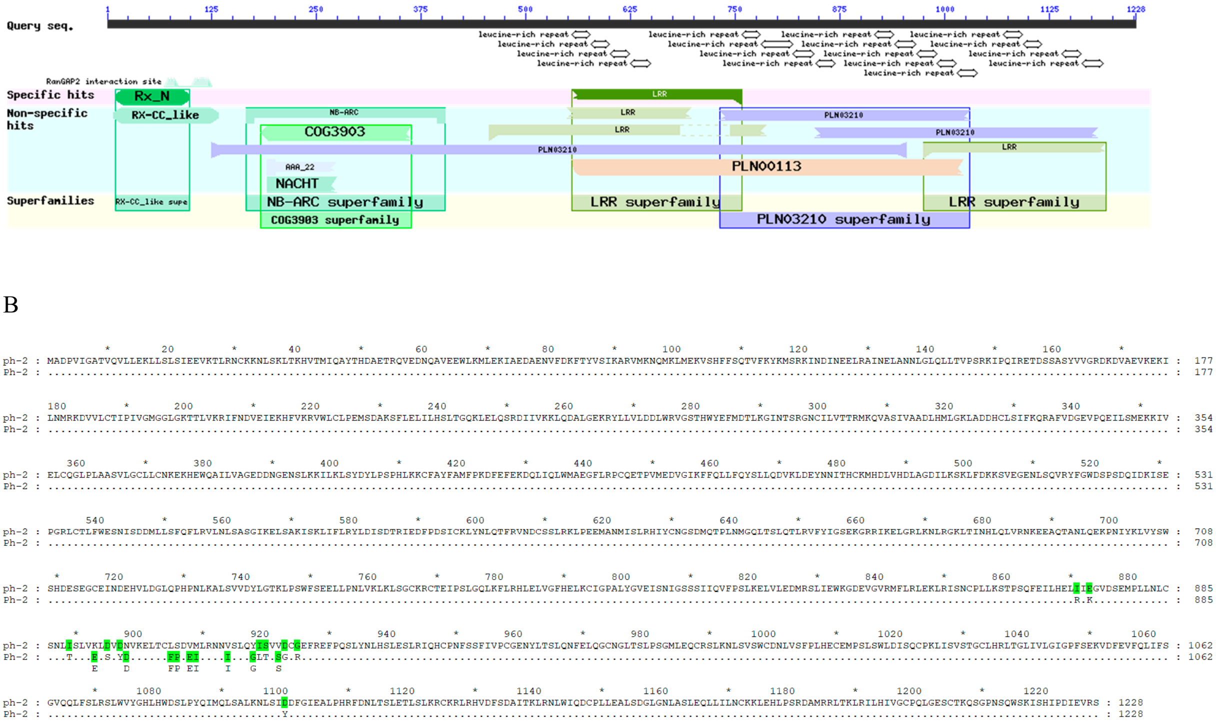

Figure S9. Structural characteristics and amino acid sequence of Ph-2. (A) Predicted structure of the protein Ph-2; (B)The alignment of the amino acid sequence between Ph-2 and ph-2.
